# Oral Levodopa Therapy, Vitamin B6 and Peripheral Neuropathy: A Cross‐Sectional Observational Study

**DOI:** 10.1002/mdc3.14243

**Published:** 2024-10-23

**Authors:** Catherine Déry, Charlie Buchmann, Geneviève Labrecque, Vicky Caron, David Simonyan, Mathieu Blais, Manon Bouchard, Nicolas Dupré

**Affiliations:** ^1^ Neuroscience Axis, CHU de Québec‐Université Laval Research Centre Québec City QC Canada; ^2^ Faculty of Medicine Université Laval Québec City QC Canada; ^3^ Neuro‐Lévis Clinic Lévis QC Canada; ^4^ Faculty of Medicine Université de Sherbrooke Sherbrooke QC Canada; ^5^ Clinical and Evaluative Research Platform, CHU de Québec‐Université Laval Research Centre Québec City QC Canada

**Keywords:** levodopa, B6, neuropathy

Peripheral polyneuropathy (PN) could affect 30–75% of Parkinson's disease (PD) patients,^1^ compared to 7–9% of older adults.^2^ Development of PN in PD is likely multifactorial and not fully understood, but may be associated with levodopa, particularly at higher doses.^1^, ^3^ Levodopa could deplete vitamin B6, B12, and folic acid, and increase homocysteine levels (Fig. S1), potentially leading to PN.^4^

A cross‐sectional observational study was conducted between May and September 2023, approved by the CHU de Québec—Université Laval research ethics committee. Fifty levodopa‐responsive parkinsonian participants with oral levodopa daily doses (LDD) ≥600 mg and disease duration ≥3 years were included. Participants with diabetes, alcohol misuse, intestinal malabsorption, PN diagnosis, or vitamin B6 supplement use were excluded.

Participants underwent clinical evaluations in the ON state using the MDS‐UPDRS and modified Toronto Clinical Neuropathy Score (mTCNS)^5^ (Tables S1 and S4, Fig. S2). Nerve conduction studies (NCS) of sensory sural and radial nerves and motor tibial and peroneal nerves from the non‐dominant side were analyzed. We obtained whole blood vitamin B6 (PLP), serum vitamin B12, serum folic acid, and plasma total homocysteine (Table S1, Fig. S3).

Statistical analyses included Wilcoxon‐Mann–Whitney tests for continuous data comparisons and Fisher exact tests for categorical data. The Box‐Cox transformation method was used for variables B6 and mTCNS for linear regressions. Correlations were estimated between predictors and electrophysiological markers of PN. Seven participants (14%) had B6 deficiency. One had low B12. PN prevalence ranged from 26.3–78%, depending on diagnostic criteria (Table S1). Using mTCNS cutoff ≥3, 78% of all participants and 73.7% of those with NCS data had PN. Univariate analysis showed LDD significantly predicted vitamin B6 levels (inverse‐transformed, *β* = 0.0069, *P* = 0.0440) (Fig. 1A and Table S2). Multivariate analysis, adjusting for age, showed LDD was not significant, but with trend towards significance (*β* = 0.0065, *P* = 0.0592) (Table S3). Higher LDD, iCOMT daily dose, and lower B6 levels (inverse‐transformed) significantly predicted worse mTCNS scores (*P*‐values: 0.0018, 0.0142, and 0.0467, respectively). Multivariate analysis showed only LDD significantly predicted mTCNS (*β* = 0.0010, *P* = 0.0068) (Fig. 1B and Tables S2, S3). Sum of nerve conduction velocities positively correlated with B6 (*r* = 0.5103, *P* = 0.0256) but not with LDD, iCOMT, age, UPDRS III, disease duration, folic acid, B12, or homocysteine (Fig. 1C and Table S2). Sum of amplitudes positively correlated with B6 (*r* = 0.4795, *P* = 0.0378) and negatively with LDD (*r* = −0.6079, *P* = 0.0058) and UPDRS III (*r* = −0.5341, *P* = 0.0185) (Fig. 1D and Table S2).

**Figure 1:**
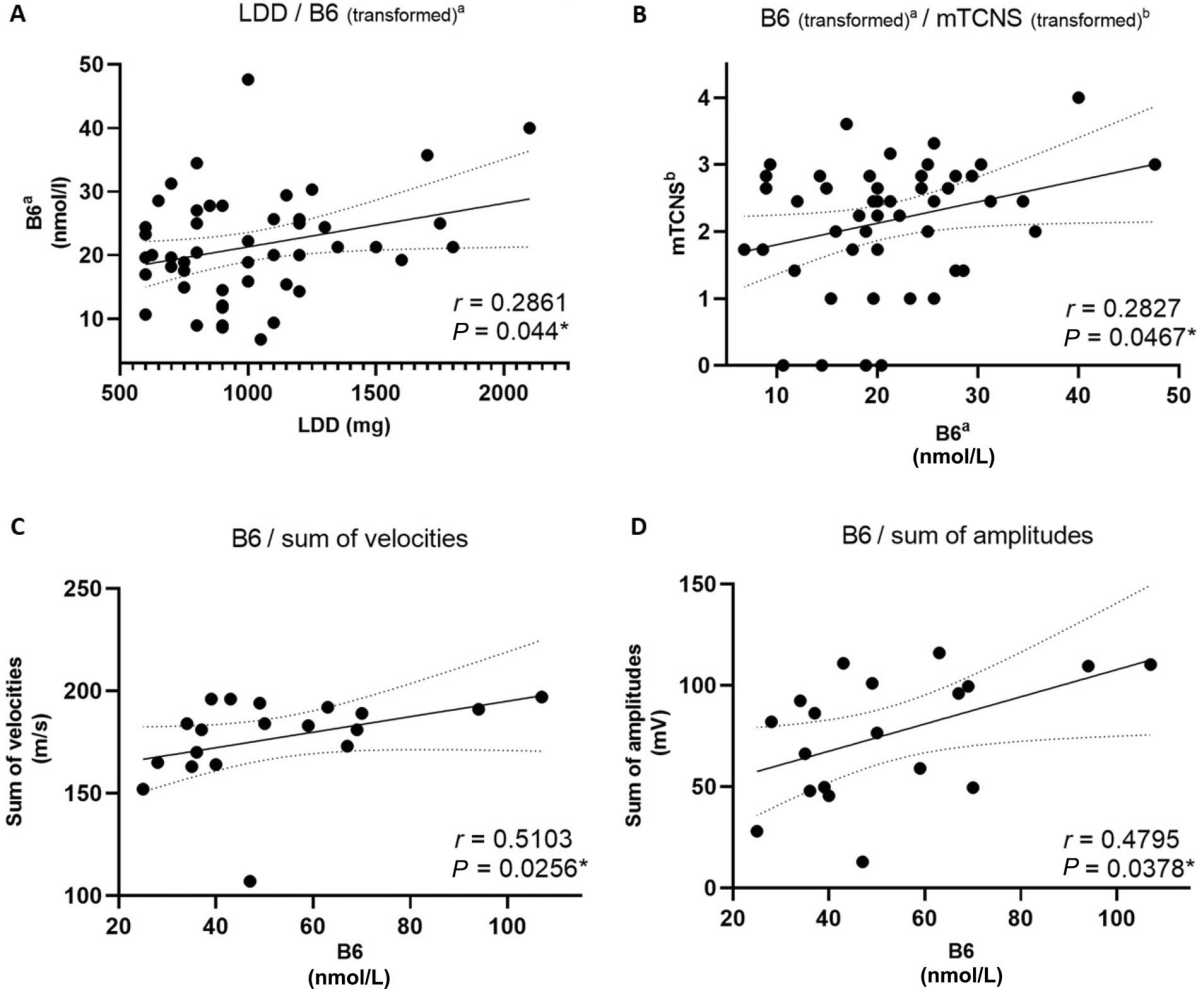
Univariate linear relations between relevant parameters. (**A**) and (**B**): transformed data for B6 and mTCNS, N = 50. (**C**) and (**D**): untransformed data, N = 19. mTCNS, modified Toronto Clinical Neuropathy Score; LDD, levodopa daily dose. ^a^ Inverse transformed B6 (1000/B6). ^b^ Square‐root transformed mTCNS. Statistical analyses were performed using SAS 9.4 and GraphPad Prism 9.5.1. A two‐sided *P*‐value ≤0.05 was considered statistically significant.

In sum, we observed relationships between higher LDD and increased PN severity, both of which were linked to lower B6 levels. Notably, B6 levels correlated with both clinical and electrophysiological markers of PN. However, Vitamin B12, folic acid, and homocysteine showed no association with PN. The design and small sample size limit causation inference and statistical power. Further research should consider large longitudinal studies to investigate B6 supplementation as a potential preventive or therapeutic measure for PN in levodopa‐treated patients.

## Author Roles

(1) Research Project: A. Conception, B. Organization, C. Execution; (2) Statistical Analysis: A. Design, B. Execution, C. Review and Critique; (3) Manuscript: A. Writing of the First Draft, B. Review and Critique.

C.D.: 1A, 1B, 1C, 2A, 2B, 3A

C.B.: 1C, 3C

G.L.: 1B, 3C

V.C.: 1C, 3C

D.S.: 2A, 2B, 3B

M.B.: 1A, 1B, 2C, 3B

M.B.: 1A, 1B, 2A, 3A

N.D.: 1A, 1B, 2C, 3B

## Disclosures

**Ethical Compliance Statement:** The present study was approved by the McGill University Health Center—neupsy‐ ethics committee and by the CHU de Québec—Université Laval research ethics committee, approval number MP‐37‐2020‐6170. Informed consent was obtained from all participants prior to enrollment. We confirmed that we have read the Journal's position on issues involved in ethical publication and affirm that this work is consistent with those guidelines.

**Funding Sources and Conflicts of Interest:** CD has received scholarships (master's program) from the Canadian Institutes of Health Research and the Fonds de Recherche du Québec—Santé. CB has received financial compensation while working on ND's team at the CHU de Québec‐Université Laval research center (internship). GL: no specific funding was received for this work. VC has received salary compensation from the Quebec Parkinson Network. DS has received salary compensation from the Quebec Parkinson Network. MB has received salary compensation from the Quebec Parkinson Network. MB: no specific funding was received for this work. ND: no specific funding was received for this work.

**Financial Disclosures for Previous 12 Months:** CD has received financial compensation while working on ND's team at the CHU de Québec‐Université Laval research center (employment). CD has also received financial compensation while working for the Faculté des Sciences Infirmières at Université Laval (employment). CB has received financial compensation while working on ND's team at the CHU de Québec‐Université Laval research center (employment). GL has received financial compensation while working at the Neuro‐Lévis clinic (employment). GL has received financial compensation while working for the Faculté des Sciences Infirmières at Université Laval (employment). VC has received financial compensation while working on ND's team at the CHU de Québec‐Université Laval research center (employment). DS has received financial compensation while working at the CHU de Québec‐Université Laval research center (employment). MB has received financial compensation while working on ND's team at the CHU de Québec‐Université Laval research center (employment). MB has received financial compensation from Abbvie, Es‐Therapeutics, Biohaven, Pfizer. ND has received a grant from the Fondation du CHU de Québec for a project entitled: Étude rétrospective sur le recours à l'aide médicale à mourir en sclérose latérale amyotrophique (SLA/AMM) au CHU de Québec‐Université Laval and a grant from Target ALS for a project entitled: Digital measurement of gait as a potential pharmacodynamic/response biomarker for ALSFTSD. He also received a grant from the CIHR for a project entitled: Unifying Molecular Omics, Multi‐Modal Neuroimaging and Artificial Intelligence for Biologically defined Patient Stratification in Amyotrophic Lateral Sclerosis and a project entitled: Depicting disease heterogeneity in neurofibromatosis type 1 and the role of microenvironment in NF1‐associated skin tumor formation through personalized tissue engineered 3D models. ND received another grant from the FRQS for the project: Quebec Parkinson's network (QPN). ND also received a grant from Alliance Santé Québec for the project Development of a sustainable health approach to research on Parkinson's and related disorders. ND received a grant from ALS Canda for the project: Crosstalk between immune response and metabolic signaling: targeting leptin/AMPK axis to restore metabolic homeostasis in ALS. ND received a grant from Brain Canada for the project Canadian Open Parkinson Network (C‐OPN). ND has a contract with The Royal Institution for the Advancement of Learning/McGill University for the project: une étude de cohorte longitudinale multi‐sites sur la maladie de Parkinson prodromique et Clinique, and the project LGMD/Pombe Registry. ND served as a consultant (reviewer) for Brain Canada. ND is part of a Scientific advisory board for the Association de la neurofibromatose du Québec and is on the Board of Director of the ARSACS Foundation. Finally, ND is employed as a Full Professor at Université Laval and as a Neurologist at the CHU de Québec—Université Laval.

## Supporting information


**Table S1.** Summary of descriptive data from the 50 participants.
**Table S2.** Univariate analysis.
**Table S3.** Multivariate analysis: multiple linear regression model of vitamin B6 and mTCNS score, final models.
**Table S4.** Comparison of patients with and without NCS data.
**Figure S1.** Levodopa metabolic pathway.
**Figure S2.** Modified Toronto Clinical Neuropathy Score recording tool used for the study.
**Figure S3.** Boxplot representation of whole blood B6 in the 50 participants, displaying median and quartiles.

## Data Availability

The data that support the findings of this study are available from the corresponding author upon reasonable request.
