# Supplementary material for: Oral Levodopa Therapy, Vitamin B6 and Peripheral Neuropathy: A Cross‐Sectional Observational Study
Source: Mov Disord Clin Pract. 2024 Oct 23;12(1):120–2. doi: 10.1002/mdc3.14243 (PMC11736868; doi:10.1002/mdc3.14243)
Supplement: Supplementary file 1 — Table S1. Summary of descriptive data from the 50 participants. Table S2. Univariate analysis. Table S3. Multivariate analysis: multiple linear regression model of vitamin B6 and mTCNS score, final models. Table S4. Comparison of patients with and without NCS data. Figure S1. Levodopa metabolic pathway. Figure S2. Modified Toronto Clinical Neuropathy Score recording tool used for the study. Figure S3. Boxplot representation of whole blood B6 in the 50 participants, displaying median and quartiles. [file MDC3-12-120-s001.docx]

**Supplementary Table 1.** Summary of descriptive data from the 50 participants

**1.1** Descriptive data

| **Parameter** (reference range/unit) | **Mean** (min-max) |
| --- | --- |
| Age (years) | 70.12 (50-83) |
| Female (%) | 16 (32%) |
| LDD (mg) | 1018 (600-2100) |
| iCOMT (mg/d) | 268 (0-1800) |
| Duration of disease (years) | 10.2 (3-30) |
| UPDRS part III score | 43.6 (14-70) |
| mTCNS score | 5.6 (0-16) |
| Hcy (4-14 umol/l) | 18.8 (9.3-44.8) |
| B12 (> 135 pmol/l) | 363.8 (83-1475) |
| Folic acid (> 8 nmol/l) | 27.8 (12.8-45) |
| B6 (35-110 nmol/l) | 55.3 (21-148) |

**1.2** Frequency distribution of blood metabolites in our participants

|  | **N (%)** | | | |
| --- | --- | --- | --- | --- |
|  | **Low^a^** | **Normal^a^** | **High^a^** | **Taking supplements** |
| **B6** | 7 (14) | 39 (78) | 4 (8) | none |
| **B12** | 1 (2) | 49 (98) | N/A | 9 (18) |
| **Folic acid** | 0 (0) | 50 (100) | N/A | none |
| **Hcy** | 0 (0) | 10 (20) | 40 (80) | N/A |

LDD = levodopa daily dose, iCOMT = catechol-O-methyltransferase inhibitors, UPDRS = Unified Parkinson's disease rating scale, mTCNS = modified Toronto Clinical Neuropathy Score, Hcy = homocysteine, N/A = not applicable

**^a^** According to our local laboratory’s reference ranges

Statistical analyses were performed using SAS 9.4 and GraphPad Prism 9.5.1.

**Supplementary Table 2.** Univariate analysis

1.1 univariate regression analysis of whole blood vitamin B6^a^

| **Predictors of B6 levels^a^** | **Parameter estimate (95% CI)** | **p-value** |
| --- | --- | --- |
| Age (years) | -0.1315 (-0.4071 to 0.1441) | 0.3421 |
| Female | -0.0686 (-5.157 to 5.019) | 0.9785 |
| **LDD (mg)** | 0.0069 (0.0002 to 0.0135) | **0.0440*** |
| iCOMT (mg/d) | 0.0024 (-0.0026 to 0.0074) | 0.3435 |

1.2 univariate regression analysis of mTCNS score**^b^**

| **Predictors of mTCNS score^b^** | **Parameter estimate (95% CI)** | **p-value** |
| --- | --- | --- |
| Age (years) | 0.0185 (-0.0125 to 0.0494) | 0.2363 |
| Female | -0.2659 (-0.8355 to 0.3037) | 0.3526 |
| **LDD (mg)** | **0.0012** (0.0005 to 0.0019) | **0.0018 **** |
| **iCOMT (mg/d)** | **0.0007** (0.0001 to 0.0012) | **0.0142 *** |
| Duration of disease (years) | 0.0168 (-0.0298 to 0.0634) | 0.4722 |
| UPDRS III score | 0.006 (-0.0133 to 0.0253) | 0.5338 |
| Hcy (umol/l) | 0.0048 (-0.0333 to 0.0428) | 0.8019 |
| B12 (pmol/l) | -0.0001 (-0.0013 to 0.001) | 0.8248 |
| Folic acid (nmol/l) | -0.0321 (-0.0645 to 0.0004) | 0.0524 |
| **B6^a^ (nmol/l)** | **0.03194** (0.0005 to 0.0634) | **0.0467 *** |

1.3 Spearman correlation between sum of **nerve conduction velocities** and other parameters (N=19)

| **Parameter** | **Coefficient (r. 95% CI)** | **P-value** |
| --- | --- | --- |
| LDD (mg) | -0.3532 (-0.7032 to 0.1346) | 0.1380 |
| iCOMT (mg/d) | -0.1740 (-0.5917 to 0.3173) | 0.4761 |
| **B6 (nmol/l)** | **0.5103** (0.05862 to 0.7886) | **0.0256 *** |
| Age (years) | -0.2848 (-0.6625 to 0.2085) | 0.2373 |
| UPDRS part III | -0.4157 (-0.7384 to 0.06194) | 0.0767 |
| Duration of disease (years) | -0.1412 (-0.5694 to 0.3472) | 0.5642 |
| Folic acid (nmol/l) | 0.1555 (-0.3344 to 0.5792) | 0.5251 |
| B12 (pmol/l) | 0.1934 (-0.2992 to 0.6046 | 0.4276 |
| Hcy (umol/l) | -0.3130 (-0.6796 to 0.1787) | 0.1920 |

1.4 Pearson correlation between­­­ sum of **nerve conduction amplitude** and other parameters (N=19)

| **Parameter** | **Coefficient (r. 95% CI)** | **P-value** |
| --- | --- | --- |
| **LDD (mg)** | **-0.6079** (-0.8323 to -0.2123) | **0.0058 **** |
| iCOMT (mg/d) | -0.1520 (-0.5671 to 0.3246) | 0.5344 |
| **B6 (nmol/l)** | **0.4795** (0.03230 to 0.7667) | **0.0378 *** |
| Age (years) | -0.3172 (-0.6742 to 0.1601) | 0.1858 |
| **UPDRS part III** | **-0.5341** (-0.7954 to -0.1055) | **0.0185*** |
| Duration of disease (years) | -0.2745 (-0.6479 to 0.2053) | 0.2554 |
| Folic acid (nmol/l) | 0.3662 (-0.1056 to 0.7034) | 0.1231 |
| B12 (pmol/l) | -0.1121 (-0.5389 to 0.3605) | 0.6478 |
| Hcy (umol/l) | -0.2334 (-0.6217 to 0.2470) | 0.3363 |

1.5 Pearson correlation between­­­ the **ratio of conduction amplitude (radial/sural)** and other parameters (N=19).

| **Parameter** | **Coefficient (r. 95% CI)** | **P-value** |
| --- | --- | --- |
| LDD (mg) | -0.3357 (-0.6854 to 0.1398) | 0.16 |
| iCOMT (mg/d) | -0.1362 (-0.5560 to 0.3390) | 0.5782 |
| B6 (nmol/l) | 0.2896 (-0.1896 to 0.6573) | 0.2292 |
| Age (years) | -0.0955 (-0.5268 to 0.3750) | 0.6975 |
| UPDRS part III | -0.2608 (-0.6393 to 0.2194) | 0.2809 |
| Duration of disease (years) | 0.0059 (-0.4495 to 0.4589) | 0.9809 |
| Folic acid (nmol/l) | -0.02651 (-0.4750 to 0.4329) | 0.9142 |
| B12 (pmol/l) | -0.02885 (-0.4768 to 0.4310) | 0.9067 |
| Hcy (umol/l) | -0.2457 (-0.6297 to 0.2347) | 0.3105 |

LDD = levodopa daily dose, iCOMT = catechol-O-methyltransferase inhibitors, UPDRS III = Unified Parkinson's disease rating scale part III, mTCNS = modified Toronto Clinical Neuropathy Score, Hcy = homocysteine

Statistical analyses were performed using SAS 9.4 and GraphPad Prism 9.5.1. A two-sided p-value ≤0.05 was considered statistically significant.

**^a^** Inverse transformed B6, (1000/B6)

**^b^** Square-root transformed mTCNS

**Supplementary Table 3.** Multivariate analysis: multiple linear regression model of vitamin B6^a^ and mTCNS score^c^, final models

| **Predictors of B6 levels^a^** | **Parameter estimate (95% CI)** | **p-value** |
| --- | --- | --- |
| Age (years) | -0.0931 -0.3642 to 0.1780 | 0.4931 |
| LDD (mg) | 0.0065 (-0.0003 to 0.0132) | 0.0592 |

| **Predictors^b^ of mTCNS^c^** | **Parameter estimate (95% CI)** | **p-value** |
| --- | --- | --- |
| **LDD (mg)** | **0.0010** (0.0003 to 0.0018) | **0.0068 **** |
| B6^a^ (nmol/l) | 0.0196 (-0.0111 to 0.0503) | 0.2051 |

LDD = levodopa daily dose, mTCNS = modified Toronto Clinical Neuropathy Score

**a** Inverse transformed B6 (1000/B6).

**b** We selected the parameters that were significant in univariate regression analysis (LD, iCOMT and inverse B6) and proceeded in a backwards fashion in multivariate linear regression analysis to select variables for the final model. Only LD remained significant when taking out iCOMT of the model.

**c** Square-root transformed mTCNS

Statistical analyses were performed using SAS 9.4 and GraphPad Prism 9.5.1. A two-sided p-value ≤0.05 was considered statistically significant.

**Supplementary Table 4.** Comparison of patients with and without NCS data

|  | **With NCS (N=19)** | **Without NCS (N=31)** |  |
| --- | --- | --- | --- |
|  | **Mean (SD)** | | **p-value^a^** |
| Age (years) | 67.6 (9.1) | 71.6 (8.7) | 0.141 |
| Female | 5 (26.3%) | 11 (35.5%) | 0.549 |
| LDD (mg) | 1080.6 (450) | 978.2 (262.1) | 0.792 |
| iCOMT (mg/d) | 368.4 (534.4) | 206.5 (433.5) | 0.217 |
| Duration of disease (years) | 9.6 (5.2) | 10.6 (6.1) | 0.515 |
| UPDRS III score | 42.2 (12.6) | 44.5 (14.9) | 0.460 |
| mTCNS score | 6.1 (4.5) | 5.3 (2.8) | 0.554 |

NCS = nerve conduction studies, LDD = levodopa daily dose, iCOMT = catechol-O-methyltransferase inhibitors, UPDRS III = Unified Parkinson's disease rating scale part III, mTCNS = modified Toronto Clinical Neuropathy Score

**a** P-value for Wilcoxon-Mann-Whitney or Fisher’s exact test statistic

Statistical analyses were performed using SAS 9.4 and GraphPad Prism 9.5.1. A two-sided p-value ≤0.05 was considered statistically significant.

**Supplementary Figure 1:** Levodopa metabolic pathway


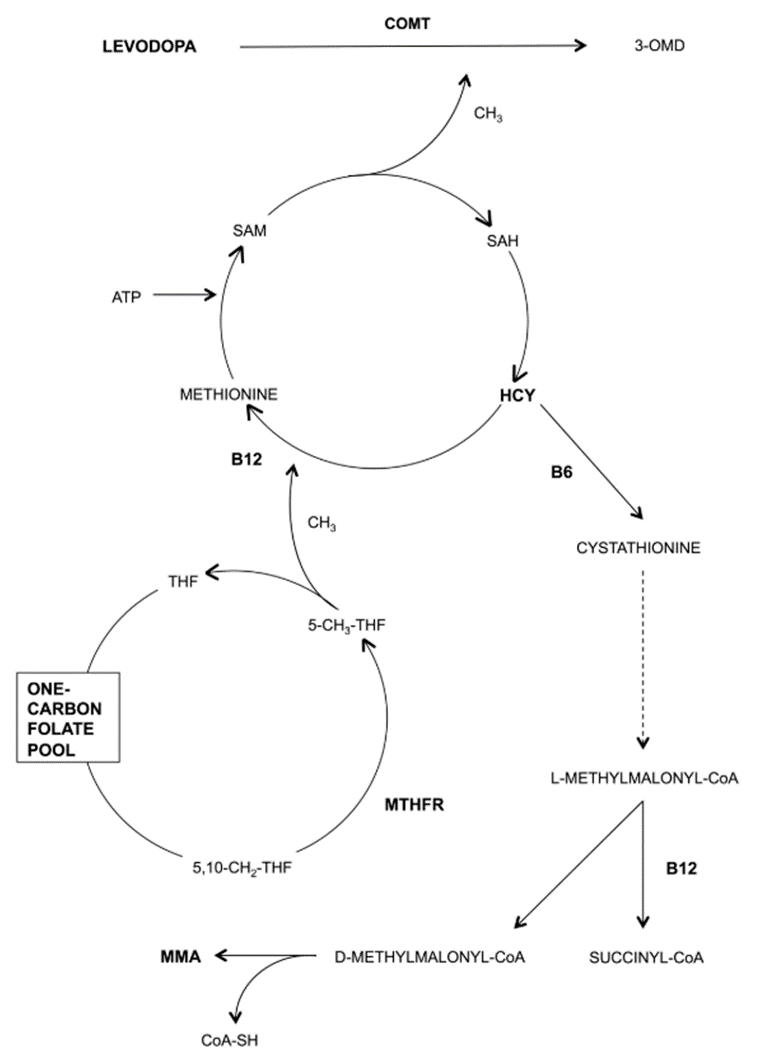


**FIG 1.** Levodopa is methylated by COMT via donation of a methyl group by S-adenosylmethionine (SAM). The reaction results in production of SAH. Then, homocysteine (HCY) is catabolized through two different metabolic pathways: remethylation to methionine, requiring 5-methyltetrahydrofolate (5-CH3-THF) as a methyl donor and vitamin B12 as a cofactor, or trans-sulfuration to cysteine, requiring vitamin B6 as a cofactor. Chronic intake of levodopa can lead to accumulation of HCY as well as depletion of vitamin B6, B12 and folate. Abbreviations: 3-OMD: 3-O-methyldopa; 5.10-CH2-THF: 5.10-methylenetetrahydrofolate. MMA: methylmalonic acid. Reused from Romagnolo A. Merola A. Artusi CA. Rizzone MG. Zibetti M. Lopiano L. Levodopa‐Induced Neuropathy: A Systematic Review. Movement Disorders Clinical Practice. 2019;6(2):96-103.

**Supplementary Figure 2.** Modified Toronto Clinical Neuropathy Score recording tool used for the study.


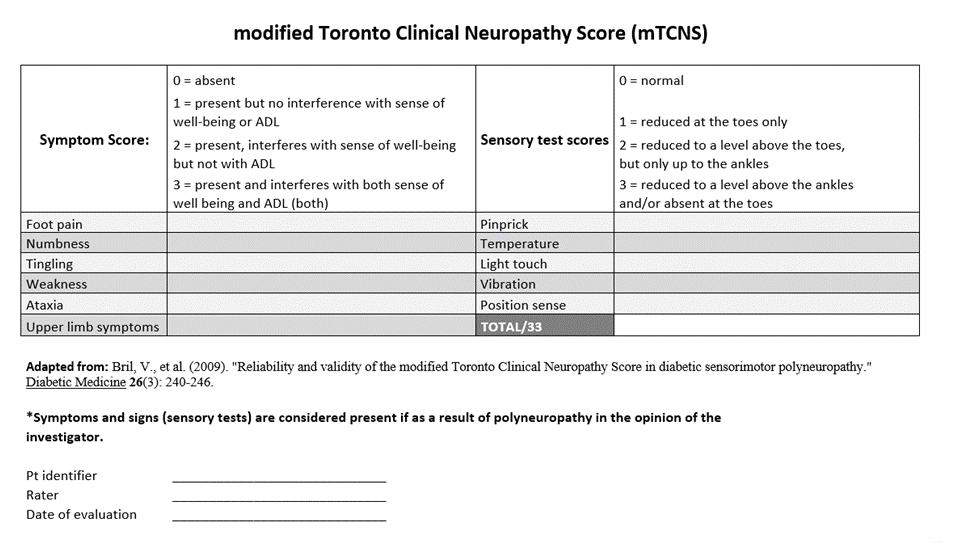


**FIG 2.** the modified Toronto Clinical Neuropathy Score (mTCNS) combines a brief semi-structured clinical interview and examination evaluating symptoms and signs of PN. The original Toronto Clinical Scoring System (TCSS) and mTCNS are validated tools for the diagnosis of PN in diabetes mellitus and have been used in similar studies of people with PD (1-2). Both tests score the severity of PN, but do not differentiate between small and large fiber PN. Creators of the mTCNS scale recommend using a cutoff score of ≥ 3 to maximize sensitivity and specificity for diagnosing PN (3).

1. Bril V, Tomioka S, Buchanan RA, Perkins BA. Reliability and validity of the modified Toronto Clinical Neuropathy Score in diabetic sensorimotor polyneuropathy. Diabetic Medicine. 2009;26(3):240-6.

2. Bril V, Perkins BA. Validation of the Toronto Clinical Scoring System for Diabetic Polyneuropathy. Diabetes Care. 2002;25(11):2048-52.

3. Idiaquez JF, Alcantara M, Bril V. Optimal cut-off value of the modified Toronto Clinical Neuropathy Score in the diagnosis of polyneuropathy. European Journal of Neurology. 2023;30(8):2481-7.

**Supplementary Figure 3.** Boxplot representation of whole blood B6 in the 50 participants, displaying median and quartiles

**
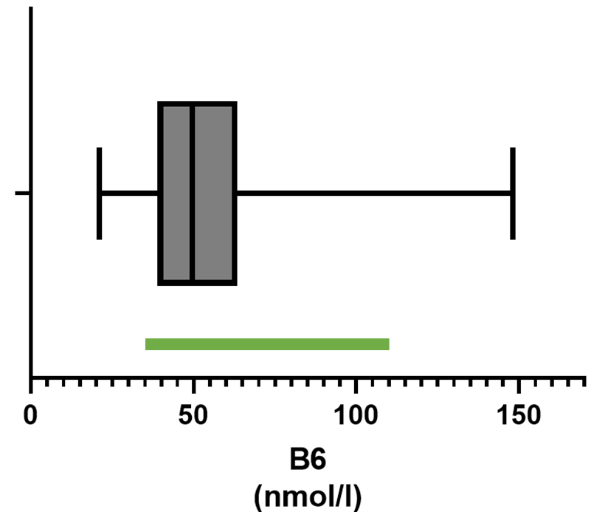
**

**FIG 3.** The thick green line represents our local laboratory’s normal reference range. At the time of this study, the platform’s laboratory evaluated B6 from whole blood. Recent data suggests that whole blood measures of vitamin B6 could underestimate deficiencies (1). Most participants from our cohort displayed whole blood B6 levels within the lower ranges of normal. Thus, the proportion of participants displaying vitamin B6 deficiency might have been higher in our cohort if B6 had been dosed in plasma instead of whole blood

1. Obeid R, Möller C, Geisel J. Circulating pyridoxal 5′-phosphate in serum and whole blood: implications for assessment of vitamin B6 status. Journal of Laboratory Medicine. 2023;47(1):23-9.
